# Hepatitis-C virus infection among injecting drug users in Lahore, Pakistan: A cross sectional study

**DOI:** 10.12669/pjms.322.9038

**Published:** 2016

**Authors:** Abdul Majeed Akhtar, Sadia Majeed, Muhammad Jamil, Abdul Rehman, Sufia Majeed

**Affiliations:** 1 Dr. Abdul Majeed Akhtar, MBBS, DTCD, DPH, MCPS, Ph.D. Department of Epidemiology and Public Health, University of Veterinary & Animal Sciences, Lahore, Pakistan. Program Manager, Provincial TB Control Program, Punjab, DGHS, 24 Cooper Road Lahore, Pakistan; 2 Sadia Majeed, M.Phil. Foodand Nutrition Department, College of Home Economics, Gulberg, Lahore, Pakistan; 3 Muhammad Jamil, M.Phil. Out Patients Medical Laboratory, Mayo Hospital, Lahore; 4 Dr. Abdul Rehman, DVM, M.Phil, Ph.D Scholar. Department of Epidemiology and Public Health, University of Veterinary & Animal Sciences, Lahore, Pakistan; 5 Sufia Majeed, Allama Iqbal Medical College, Lahore, Pakistan

**Keywords:** Injecting drug users, Viral load, ELISA kits, HCV infection

## Abstract

**Objectives:**

To determine the prevalence and risk factors of hepatitis C virus among injecting drugs users, furthermore different genotypes of HCV infection and their effect on viral load were also found and subsequently most prevalent subtype was predicted.

**Methods:**

All samples were processed for Anti-HCV antibody detection through ELISA by using third generation ELISA Kit. The Anti-HCV positive serum samples were stored for RT-PCR to estimate the viral load and genotypes of HCV for study. Injecting drug users selected from in and around Lahore Metropolitan from July 2012 to August 2013 was included. The data analysis was completed by using SPSS version 16. A p-value of < 0.05 was considered to be significant.

**Results:**

A total of 241 Injecting drug users were enrolled and screened for Anti HCV in the study. Prevalence of HCV infection in IDUs from Lahore was found to be 36.09%. Education (p=0.000), low socioeconomic status (p=0.011), Blood transfusion (0.003), any tattoo on the body (p=0.002), use of injectable drugs with reused syringes (p=0.000) and sharing of syringes (p=0.001) in groups was significantly associated with HCV infection. Some utensils were also significantly associated with HCV status. The most common subtype of HCV genotype was 3a (n=65) followed by 2a (n=15) and 1a (n=6).

**Conclusion:**

The study reveals that IDUs with reused syringes status and sharing of syringes in group had more chances to get HCV infection. The viral load in IDUs infected with different subtypes of genotype was significantly associated.

## INTRODUCTION

It was observed that prevalence of HCV in Pakistan was 57±17.7% among IDUs.^1^ In China, USA and Russia 1.6, 1.5 and 1.3 million people were affected by HCV.^2^ The risk factors associated with HCV infection were, injecting for more than three years, injecting with person suffered with hepatitis, sexual heterogenicity and with sharing cotton.^3^ Injection drug users (IDUs) were at risk of getting hepatitis C virus infection. The decline of prevalence of HCV was due to declines in injection equipment sharing practices, condom use, and hepatitis B vaccination.^4^-^6^

There was a high prevalence of HIV and HCV among injecting drug users (IDUs). A study was conducted in Lahore and Quetta in 2003 on 351 IDUs. No case of HIV was observed, while HCV seroprevalence was 88%. The awareness about HCV was low. Injection behaviors and receiving a street barber shave were significantly associated with HCV seropositivity.^7^ Injecting drug users, co-morbid mental health conditions, poor socioeconomic status and a complex treatment protocol incompatible with the life styles of IDUs had made poor uptake and incompletion of HCV treatment.^8^,^9^

Hepatitis C virus was transmitted because of reusing syringes and using single-use medication vials on multiple patients.^10^,^11^ There was a high prevalence of HCV found, among non-injecting drug users, smokers, heroin, cocaine, crack, or methamphetamine users.^12^ A study was conducted in Iran on 499 prison inmates to determine the HCV prevalence among them. Through physical examination and by urine collection it was determined that they were IDUs. HCV prevalence was 80.0%, factors associated with HCV infection were, history of prison, age of first injection and tattooing. It was found that incarceration was responsible for the spread of HCV.^13^,^14^

Ever having a tattoo and having intravenous and intramuscular injections in the past 12 months were marginal risk factors for HCV (p-values < 0.01) among the blood donors.^15^,^16^ The objectives of the present study were to estimate the prevalence of Hepatitis C Virus infection among IDUs diagnosed with anti HCV antibody in and around Lahore Metropolitan. It also discussed the various demographic and risk factors associated with HCV infection. The different genotypes of HCV were also detected and their effect on viral load was determined in this study and prevalent subtype was predicted.

## METHODS

### Data

The initial data collection was done by convenient sampling then snow ball sampling technique was used to recruit more participants and data collection was done through a pre-designed questionnaire (to study the risk factors associated with HCV infection) from July 2012 to August 2013. Injecting drug users who refused to fill the questionnaire or were reluctant to give blood samples were excluded from the study. In this study 241 injecting drug users from Lahore were included. Present study was carried out to estimate the prevalence of laboratory confirmed Hepatitis-C virus infection in injecting drug users among the population of Lahore metropolitan. The risk factors of Hepatitis-C virus infection in injecting drug users in and around Lahore Metropolitan were also assessed. Subsequently distribution of genotypes of Hepatitis-C virus among injecting drug users through RT-PCR was assessed. With informed and written consent, venous blood was collected and serum was separated for ELISA. All samples were processed at Mayo hospital Lahore for Anti-HCV antibody detection through ELISA and third generation ELISA Kit (ETI-AB-HCVK-4, Diasorin S.P. A Italy) containing 96 wells was used for Enzyme Linked Immunosorbent Assay (Rebuzzini, 2008). The Anti-HCV positive serum samples were stored and processed for RT-PCR for HCV RNA Quantification to check the viral load. Among sample with detectable viral load (i.e >500iu) were further processed for genotyping by Real-time PCR Cepheid smart cycler which was applied by using QIAamp Mini column kit and Sacace HCV Genotyping kit.

### Statistical data analysis

All the quantitative data was presented in the form of frequency, percentage and mean ± S. D. The qualitative data was presented in the form of proportion and percentage where appropriate. T-test was used to access the difference of means between reactive and non-reactive injecting drug users. A P-value < 0.05 was considered to be significant. Odds Ratio with 95% Confidence interval was used to see the magnitude of dependency on various risk factors like demographic characteristics, type of drugs, type of syringes, blood transfusion and donation status and effect of sharing utensils etc.

### Statistical software

The data was analyzed statistically by using SPSS version 16.^17^

## RESULTS

In this study 241 injecting drug users were enrolled and tested for Anti HCV. Among the selected injecting drug users 87 (36.09%) were reactive. Mean age of reactive and non reactive injecting drug users was 33.39±11.19 and 31.22±11.99 years. There was non-significant difference between ages of reactive and non-reactive injecting drug users i.e. (P= 0.618) (Table-I).

**Table-I:** Distribution of reactive (Positive) & non-reactive (Negative) injecting drug users according to age.

	Anti Hepatitis C Virus		Total	P-value

	Reactive	Non-Reactive		
Number (%)	87 (36.09%)	154 (63.91%)	241	0.618
Mean (Age)	33.39	31.22	32.00	
Std. Deviation	11.19	11.99	11.73	

Gender distribution for reactive injecting drug users was 86 male and one female. Qualification and socioeconomic status were significantly associated with Anti-HCV status (p=0.000) while Gender, ethnicity, marital status, occupation, income per month were insignificantly associated with Anti-HCV status. All these injecting drug users used injectable drugs and all these drug users were taking injectable drugs for the time duration between 1-3 years. Among these injecting drug users no one had under gone any dental procedure, injury treatment and ever sent to lock up. Sold blood was not reported by any of these injecting drug users. Sexual relation other then wife was not reported by any of these injecting drug users. Types of syringes (p=0.000), usage of drugs (single/group) (p=0.001), blood transfusion (p=0.003) and tattoo on the body (p=0.002) were significantly associated with Anti-HCV status. Ever involved in commercial sex activity, how do these injecting drug users shave and disinfecting the ustra was insignificantly associated with Anti-HCV status for these drug users (Table II & III).

**Table-II:** Distribution of hepatitis c virus reactive & non-reactive injecting drug users according to demographic characteristics.

Demographic Characteristics		Anti Hepatitis C Virus		p-value	ODDS Ratio	Confidence Interval

		Reactive	Non Reactive			
Gender	Male	86	154	-	-	-
	Female	1	0			
Geographical Status	Punjab	84	151	0.472	0.5563	0.10-2.817
	Non Punjab	3	3			
Marital Status	Married	31	54	0.929	0.975	0.563-1.690
	Unmarried	56	100			
Educational Status	Illiterate	80	105	0.000	5.333	2.294-12.40
	Educated	7	49			
Occupational Status	Public Job	17	18	0.096	1.835	0.890-3.78
	Private Job	70	136			
Socioeconomic Status	5000-10000	87	152	0.011	2.869*	0.13-60.43*
	>40000	0	2			

* Odds Ratio was calculated by adding 0.5 in each cell.

**Table-III:** Summary of association between Hepatitis-C and various indicators about injecting drug users.

Indicators	Response	Anti Hepatitis C Virus		p-value	ODDs Ratio	Confidence Interval	

		Reactive	Non-Reactive			Lower	Upper
Type of drugs you use	Injectable	87	154	-	-	-	
Duration of drug Use	1-3 Years	87	154	-	-	-	
Type of syringes	Used	71	89	0.000	3.241	1.727-6.083	
	New	16	65			
How do you use drugs	Single	35	98	0.001	0.408	0.237-0.703	
	Both (Single+Group)	49	56				
Have you ever been sent to Prison	Yes	1	1	0.681	1.779	0.109-28.8	
	No	86	153				
Dental procedure	No	87	154	-	-	-	
Sexual contact other then wife	No	87	154	-	-	-	
Any tattoo on the body	Yes	70	93	0.002	2.701	1.452-5.024	
	No	17	61				
Injury Treatment	Self	86	153	0.681	1.779	0.110-28.802	
	Local Dispensary	1	1				
Blood Transfusion	Yes	56	68	0.003	2.285	1.329-3.928	
	No	31	86				
Blood Donation	Yes	60	99	0.462	1.235	0.704-2.164	
	No	27	55				
Ever sold blood	No	87	154	-	-	-	
Commercial sexual activity	Yes	1	1	0.681	0.562	0.035-9.100	
	No	86	153				
Cleanliness of the Barber shop	Bad	87	154	-	-	-	
How do you shave	Self	86	153	0.681	1.779	0.110-28.802	
	Barber	1	1				
Disinfect Ustra	Yes	86	153	0.681	1.779	0.110-28.802	
	No	1	1				
Do you have any relative having	Hepatitis	-	-	-	-	-	
	Non Hepatitis	-	-				
	No Disease	87	154				

Comb, spoon, towel and nail cutter sharing was reported by all the reactive and non reactive injecting drug users. While most of the reactive injecting drug users told that they shared tooth brush (p=0.000, OR= 4.81) and razors (p=0.001, OR=6.09) with others which had significant affect on the Anti-HCV status of these IDUs. Glass sharing and straw sharing was insignificantly associated with Anti-HCV status (Reactive/Non-Reactive). i.e. [Glass Sharing (p= 0.681, OR=1.779), Straw sharing (p= 0.559, OR=1.788)] (Table-IV).

**Table-IV:** The effect of sharing utensils and other items by injecting drug users.

S.N.			Anti Hepatitis C Virus		p-value	ODDs Ratio	Confidence Interval

			Reactive	Non Reactive			
1	Comb Sharing	Yes	87	154			
		No	-	-			
2	Glass Sharing	Yes	86	153	0.681	1.779	0.110-28.802
		No	1	1			
3	Spoon	Yes	87	154			
		No	-	-			
4	Towel Sharing	Yes	87	154			
		No	-	-			
5	Straw Sharing	Yes	85	152	0.559	1.788	0.247-12.924
		No	2	2			
6	Razor Sharing	Yes	68	57	0.001	6.09	3.327-11.15
		No	19	97			
7	Nail Cutter	Yes	87	154			
		No	-	-			
8	Tooth Brush	Yes	78	99	0.000	4.815	2.241-10.34
		No	9	55			

Table-V summarizes the distribution of HCV genotypes and subtypes of HCV genotypes in injecting drug users among the positive cases of the study. Among 87 patients reactive for Anti-HCV, 06 patients’ viral genotype was Type-1, 15 patients’ viral genotype was Type-2, 65 patients’ viral genotype was Type-3 and 01 patients’ viral genotype was not detected. The most prevalent subtype of HCV genotype was 3a (n=65) followed by 2a (n=15) and 1a (n=06).

**Table-V:** Distribution of HCV genotypes (Subtypes) in injecting drug users.

Types of HCV Genotypes							

	Type-1	Type-2	Type-3	MG*	ND**	UT***	Total
Injecting Drug Users	6(1a)	15(2a)	65(3a)	0	1	0	87

* MG= Multiple Genotypes,

** ND= Not detected,

*** UT= Un-typeable

Table-VI summarizes the HCV RNA Viral Load (iu/ml) with respect to its sub types in injecting drug users. The viral load in injecting drug users infected with sub types of HCV genotype 1a was highest followed by 2a and 3a. Viral load among different sub types of HCV genotypes was significantly different (p=0.003) in injecting drug users.

**Table-VI:** Study of HCV RNA viral load (IU/ml) with respect to its sub types in injecting drug users.

Group	Genotype	Number	Mean	Std. Deviation	p-value
I.D Users	1a	6	10158889.33	12540518.875	0.003
	2a	15	5675984.67	10084255.199	
	3a	65	1454195.66	3761904.659	
	Not Detected	1	250.00	-	

Total	87	2765702.00	6550510.018

## DISCUSSION

The prevalence of HCV infection among IDUs in this study is 36%. Prevalence of HCV is most frequent in injection drug users (IDUs) and it ranges from 27% to 93%.^5^ The results of other studies may be due to difference in study design as well as difference in the era of their conduction. Another study conducted elsewhere has shown poor socio economic conditions for causing HCV infection. Their results are similar with our study as all of the HCV Positive persons have poor social economic condition.^9^ It is evident that the risk factor like IDUs, unsafe sexual activities, tattooing & unhygienic having equipments responsible for causing HCV injection.^11^ The risk factor they observed are consistent with the risk factors that we observed in our study. A study in Iran found HCV prevalence 80%.^13^ Their results are consistent with our study because of difference in the demographic regions as well as the ethnicity.

This range is due to the duration of IDU in respective population. According to Tseng, HCV prevalence is 66.2% in patients who were using injecting drugs for more than 9 years, 87.6% for duration of 10-19 years, 97.6% for 20-29 years of duration and 98.7% for >30 years.^18^ In our study we evaluated 241 injecting drug users and the HCV prevalence was found to be 36.09% which is similar to the above mentioned studies. We also demonstrated that the majority of HCV positive patients were males, illiterate and unmarried.

The majority of HCV positive persons were self employed & their monthly income was less than 10,000 rupees. All of the HCV positive persons were using injection for the last three years & had tattoos on their bodies. All of them had dental procedures and had extra marital relationship. All of HCV positive person involved in blood transfusion and blood donation. All of them except one were in the habit of making shaves by themselves and none of their relative ever had HCV, HBV and HIV. This indicates that 32% HCV prevalence which is almost similar to our study. They also indentified IDUs at high risk of getting HCV, HIV and HBV infection. Another study conducted in Lahore and Baluchistan among the IDUs found 88% of HCV Prevalence which is similar to our study but equal to the reports of CDC.^19^

The two very important predictive indicators, hepatitis C virus genotype and viral load, play a significant role in making treatment decisions. It has been observed that severity of the HCV infection, its progression and response to anti-viral therapy may vary according to the genotype. In this study were veiled that 3 a subtype of genotype was most common among IDUs in Lahore followed by 2a and 1a. These findings of the present study were similar with other study carried out in Pakistan reporting 3a subtype of genotype as most predominant genotype.^20^ It was observed that the viral load was highest in 1a as compared to 2a and 3a but the prevalence was lowest in 1a as compared to 2a and 3a which indicate that high viral load increased the severity of the disease and decreased the life span of the individual.^21^
